# Loop Electrosurgical Excisional Procedure (LEEP) Done for Discrepancy: Does the Time from HGSIL Affect Pathologic Grade of CIN in LEEP Specimen?

**DOI:** 10.1155/2010/743097

**Published:** 2010-06-29

**Authors:** Sue L. Moreni, Caroline M. Mitchell, Rochelle L. Garcia, Linda O. Eckert

**Affiliations:** ^1^Department of Obstetrics and Gynecology, University of Washington, Seattle, WA 98195-6460, USA; ^2^Department of Pathology, University of Washington, Seattle, WA 98195-6460, USA

## Abstract

*Objective*. When pathologic discrepancy arises between high-grade cytology on Papanicolaou (Pap) smear and low-grade histology on cervical biopsy, Loop Electrosurgical Excisional Procedure (LEEP) is one management alternative. Our objective was to determine whether the time from initial HGSIL Pap to LEEP affects the pathologic grade of the LEEP specimen. *Study Design*. We performed a retrospective case-control study identifying LEEPs performed for discrepancy over a 10-year period (1997–2007). 121 subjects were separated into two groups based on LEEP pathology (≤CIN 1 and CIN 2,3) and compared using *χ* ^2^. *Results*. Of the 121 LEEP specimens, 67 (55.4%) had CIN 2,3. CIN 2,3 was more often discovered when LEEP was performed within 3 months of the HGSIL Pap smear versus after 5 months (55.2% versus 16.4%, *P* = .096). *Conclusion*. Women undergoing LEEP for discrepancy >5 months from their HGSIL Pap demonstrated a trend toward less CIN 2,3 on LEEP pathology.

## 1. Introduction

Women with high-grade Papanicolaou (Pap) smears have a 43%–66% risk of having moderate- to high-grade intraepithelial neoplasia (CIN 2 or CIN 3) on subsequent biopsy and a 2% risk of having invasive cancer [1]. However, when discrepancies occur between Pap smear cytology and cervical biopsy histology, this can cause a clinical dilemma. We define discrepancy as patients with High-Grade Squamous Intraepithelial Lesion (HGSIL) Pap smear followed by cervical biopsies with Cervical Intraepithelial Neoplasia (CIN) 1 histology or less. In cases of discrepancy, the American Society for Colposcopy and Cervical Pathology (ASCCP) previously favored an excisional procedure for diagnosis in nonpregnant patients when no lesion or CIN1 is identified with satisfactory colposcopy [2]. As we develop a better understanding of HPV infection, its clinical course, and prognosis, more people are considering conservative management. The most recent ASCCP guidelines in 2006 included the option of repeating Pap smear and colposcopy every 6 months for 1 year in women over 20 with discrepancy and recommended this conservative monitoring pathway in the adolescent population [3].

In nonadolescent patients, a loop electrosurgical excisional procedure (LEEP) is a reasonable management option for discrepancy, as there is a concern that the high-grade lesion found on Pap smear was missed on the colposcopically directed biopsy. However, we know that as many as 35% of women with HGSIL will regress spontaneously, making the excisional procedure unnecessary [4]. Data evaluating the time from the initial HGSIL cytology to the LEEP, as it impacts likelihood of finding significant pathology in the LEEP specimen, has not been well characterized. Our objective was to determine whether the time from initial HGSIL Pap to LEEP, when done for discrepancy, affects the pathologic grade of CIN in the LEEP specimen. We hypothesized that, as the time interval between the initial HGSIL Pap and subsequent LEEP for discrepancy increased, the likelihood of finding CIN 2-3 in the LEEP specimen would decrease.

## 2. Materials and Methods

This was a retrospective case-control study of all LEEP procedures done for discrepancy at the University of Washington Medical Center and Harborview Medical Center in Seattle, Washington. We identified potential cases by performing a search in the pathology database to identify all LEEP specimens between January 1, 1997 and August 31, 2007. Of note, the Dysplasia Clinics at these two medical centers serve as referral centers for women diagnosed with abnormal cytology at local clinics. Thus, not all initial Pap smears originated from these two medical centers. In the two Dysplasia Clinics, a standardized colposcopy form is used to record impression and results of colposcopy, but the Pap smear is not routinely repeated, nor do we have a complete history of prior Pap smears. Based on colposcopic findings, biopsies were performed at the discretion of the attending physician. 

To determine which LEEPs were done for discrepancy, we linked all LEEPs with their respective preceding Pap smear, colposcopy report, and cervical biopsy result. We included all women, 18–49 years old, who had pathologic discrepancy as defined by HGSIL Pap smear and a subsequent colposcopy with cervical biopsies of CIN 1 or less. We excluded those who had an unsatisfactory colposcopy exam, previous excisional procedure, and positive endocervical curettage and those who were pregnant or HIV positive. 

Once discrepant cases were identified, we performed a chart review and used a standardized data collection instrument to gather general demographic information and risk factors associated with progression of cervical neoplasia (smoking, number of partners, age of coitarche, history of sexually transmitted infections, and birth control method). On this form we also documented cytologic and histologic results of the Pap smear, cervical biopsy, and LEEP procedure, and recorded the length of time between the Pap smear and the LEEP. 

A power calculation was based on the assumption that, in LEEPs done for discrepancy, the prevalence of CIN 2 or 3 found in the LEEP specimen will be higher when the LEEP was performed closer to the time of the HGSIL Pap. We divided the time intervals from Pap smear to LEEP into three intervals: less than three months, between three and five months, and greater than five months. As this was a retrospective study, we used our data of 55% prevalence of CIN 2,3 in LEEP specimens when the time interval from the Pap smear to the LEEP is less then 3 months and 16% when the interval is greater than five months. From this, the calculated sample size was 23 women to give 80% power and *α* = 0.05 to detect this difference in pathologic result, demonstrating that the study was appropriately powered to test our hypothesis.

Based on their LEEP histology, subjects were separated into two groups: CIN 1 or less and CIN 2,3. The “CIN 1 or less” group included normal, cervicitis, and CIN 1. The “CIN 2,3” group included CIN 2 and CIN 3. We chose these two groups based on clinical application, as this is the general division that determines treatment management. Statistical analysis was executed with SPSS v.16. Differences between the two groups were examined using Student's *t*-test and *χ* ^²^. Univariate and multivariate logistic regressions were used to calculate odds ratios and 95% confidence intervals for the association between time since HGSIL and histologic grade of LEEP. 

This study was approved by the International Review Board at the University of Washington, IRB Application Number 07-8885-E/A 01.

## 3. Results

Of the 1,356 patients who underwent a LEEP during this time period, 157 were performed for discrepancy. Of these, 36 women were excluded: 24 had unsatisfactory colposcopy, six had endocervical curettings positive for neoplasia, four were HIV positive, and two were older than 49. The 121 remaining patients were divided into two groups based on the pathologic grade of CIN in their LEEP specimen (CIN 1 or CIN 2,3). As demonstrated by Table 1, the two groups were similar with regard to age, ethnicity, parity, tobacco use, coitarche, number of partners, and history of sexually transmitted infections. There was a difference noted in the use of birth control method, likely due to the number of Depo-Provera users in the CIN 2,3 group.

**Figure 1:**
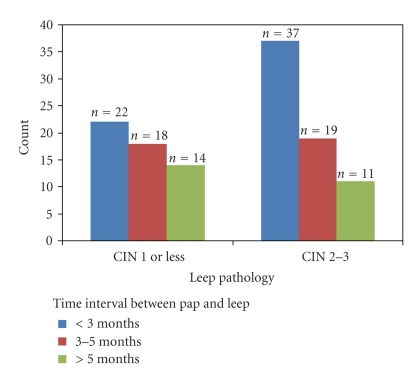
Subjects were divided into two groups by their LEEP histology (CIN 1 or CIN 2,3), and we examined the time interval from their HGSIL Pap to their LEEP. The blue bars signify those subjects who had their LEEP done less than 3 months from the HGSIL Pap. The red bars are those who had their LEEP 3–5 months from the HGSIL Pap. The green bars are those who had their LEEP after 5 months of the HGSIL Pap.

Of the 121 patients who underwent a LEEP for discrepancy, 67 patients (55.4%) had CIN 2,3 on their LEEP pathology specimen and 54 (44.6%) had CIN 1 or less. We examined the time interval from the initial HGSIL Pap smear to the LEEP procedure in the CIN 2,3 group. Of the 67 who had CIN 2,3 on LEEP histology, 37 (55.2%) had their LEEP within three months of the initial Pap versus 11 (16.4%) who had their LEEP greater than five months after the Pap smear (Figure 1). Although there was no significant difference in the time interval between the groups, the Pearson correlation coefficient, a measure of linear trend, was −0.15 (*P* = .096). Though not statistically significant, this suggests a trend toward decreased prevalence of CIN 2,3 in the LEEP specimen as the time from HGSIL Pap smear increased. In order to explore this further, a univariate logistic regression was performed to test for trend of LEEP histology over time using CIN 2,3 as the outcome (Table 2). In this regression as time interval increased, the likelihood of CIN 2,3 decreased, though the effect did not reach statistical significance.

Although it was not our initial intent to examine the effect of birth control method on the histologic grade of the LEEP specimens, there was a significant difference in the use of Depo-Provera between women with low-grade and moderate- or high-grade CIN (3.8% in CIN 1 versus 15.2% in CIN 2,3). We therefore performed a multivariate regression using the covariates of time interval and birth control method to analyze the effect of birth control method on dysplasia (Table 3). Birth control method was divided into three groups: nonhormonal users (no method, tubal ligation, barrier methods, and Copper IUD), estrogen and progesterone combined methods (oral contraceptive pills, NuvaRing, patch), and progesterone only methods (Depo-Provera). Depo-Provera users were more likely to have CIN 2,3 in their LEEP specimen (OR 7.59, *P* = .02).

Because of this significant relationship, we performed the multivariate regression in the CIN 2,3 group controlling for contraceptive method. After controlling for the birth control variable, we demonstrated a trend toward fewer CIN 2,3 LEEP findings when the procedure was performed more than five months from the HGSIL Pap, a trend that neared but did not achieve significance (OR 0.39, *P* = .07).

## 4. Discussion

This study was initiated prior to the 2006 ASCCP Consensus Guidelines, which were released in October 2007. New changes announced in the guidelines included those that direct the management of abnormal cytology in adolescents of 20 years and younger. For adolescents in the discrepant situation, it is now recommended that they be observed and monitored with Pap smears and colposcopy examinations every 6 months for 24 months [3]. Conservative management in this group has been favored, as we know that the majority of HPV infections will clear without treatment within two years and have negligible long-standing clinical significance [5]. In addition, although generally well tolerated, LEEPs are not without complications, including risks to future pregnancies such as premature delivery, premature rupture of membranes, and low birth weight [6–8]. We did not examine adolescents separately in this study as there were only 14 subjects that were 20 years or younger at the time of their LEEP. However, our results suggest that the cautious approach in treating adolescents that the ASCCP is now advising is not detrimental. 

The role of exogenous estradiol and progesterone in the development of cervical cancer has been discussed extensively. There is still insufficient data as to whether oral contraceptive pills are associated with the development of cervical cancer in HPV-infected women [9]. Thomas et al. showed an increase risk of CIN 3 but not invasive carcinoma in Depo-Provera users, a risk that increased with the length of use [10]. Many have examined the immunohistochemical presence of estrogen and progesterone receptors within the cervix and compared the makeup of receptors in normal cervix with those that contain neoplasia [11]. Konishi et al. have suggested that HPV infection may increase progesterone receptor expression that is found in neoplastic cervical squamous cells [12]. Progesterone has been hypothesized to be a modulator of the immune system [13]. In this way, the exposure to Depo-Provera may decrease the clearance of HPV infection. However, the use of hormonal birth control was not the focus of this study, and as a result this study was not powered to examine this association. Still, this is an intriguing finding that may merit further investigation. 

One limitation of our study is that the University of Washington and Harborview Medical Centers are referral centers for cervical neoplasia. As many patients are referred from providers outside the system, original Pap smears are not always available to us to confirm the HGSIL nor is the Pap smear routinely repeated at the time of colposcopy. Studies demonstrate that pathologists vary in their determination of cytologic grading of Pap smears [14]. If there is a tendency to err on side of overcalling the Pap smear HGSIL and the patient has a colposcopy consistent with CIN1 or less, this would lead to discrepancy. A LEEP done in this scenario would likely have lower-grade CIN than in a situation where a pathologist is more likely to read LGSIL on the original Pap smear. 

Another limitation of this study is the inherent disadvantage of inter observer variation on colposcopy examination because, in our clinics, multiple providers perform colposcopy. We know that colposcopy is imperfect and up to 33% of CIN 2,3 can be missed on a single colposcopy examination [15]. Missing the diagnosis of CIN 2,3 on colposcopy and cervical biopsies after an HGSIL pap smear places the patient in a discrepant situation and previously obligated them to a LEEP. However, this would likely have given us a higher number of CIN 2,3 on LEEP histology, as it may have been present but missed on the preceding colposcopy examination.

In addition, we used medical records of LEEP pathology to identify cases of discrepancy. This may open the potential of selection bias as some cases could be missed, or inadvertently miscoded. These LEEPs were identified, though, as a pathology database, and Table 1 shows that our two groups were similar. 

Finally, our examination of birth control method as it affected the LEEP pathology was not adequately investigated. There are many subtle effects of birth control on cervical dysplasia. We grouped non-hormonal methods together, when in fact this may represent a heterogeneous group of women. Women who are using no method may or may not be abstaining from intercourse. Those that are abstaining may have less exposure to HPV, which could lead to a higher rate of HPV regression. In the same way, we would expect condom users to have less exposure to HPV as Winer et al. have shown that condoms prevent HPV transmission [16]. A separate study would be required to elucidate the role of all the various birth control methods as they influence cervical dysplasia discrepancies over time.

## 5. Conclusions

To our knowledge, this is the first study that examines the effect of time on histologic grade of CIN in the LEEP specimen when LEEP is performed for discrepancy. Research demonstrates that the neoplasia caused by HPV may regress spontaneously with time [4, 5]. Hence, we sought to determine whether the length of time between the initial HGSIL Pap smear and a LEEP performed for discrepant pathology on cervical biopsy affects the pathology results of the LEEP specimen. We demonstrated a trend suggesting that, when LEEP is performed more than 5 months from the initial HGSIL Pap smear, isolating CIN 2,3 in the LEEP specimen is less likely. This trend was more pronounced in the multivariate regression controlling for birth control method, suggesting that birth control method may impact regression of HPV. When controlling for birth control method, the trend toward decrease in CIN 2,3 with time was more clear as there were more women using Depo-Provera who had CIN 2,3 and had their LEEP greater than five months from their HGSIL Pap smear. 

Many have explored the option of “See-and-Treat” for HGSIL management with immediate LEEP in recent years. The 2006 ASCCP Guidelines advocate caution and observation rather than an immediate excisional procedure in women who are still considering childbearing due to the increased pregnancy complications [3]. We cannot directly extrapolate our data into the risk of overtreatment with immediate LEEP. Whenever colposcopy is performed, one must also take into account the immunological stimulation that is thought to accompany colposcopic biopsy of cervical neoplasia that hastens resolution [17]. In this study alone if all patients went to an immediate LEEP, at most 45% of women may have had CIN 1 or less on LEEP and would have been overtreated. Some studies have shown a much lower overtreatment rate when the time interval from Pap to LEEP is diminished [18, 19]. However, it would be difficult to advocate for immediate LEEP in our patient population based on the percentages of this study. Given the propensity of HPV clearance over time, a “see-and-treat” approach deserves scrutiny. This study suggests that, when in a discrepant situation, the variable of time between the initial HGSIL Pap and LEEP may be a consideration in determining whether a LEEP is appropriate.

## Figures and Tables

**Table 1:** Demographic characteristics comparing women with CIN 1 or CIN 2,3 on LEEP.

Characteristic		CIN 1 or less	CIN 2-3	*P*
		*n* = 54 (%)	*n* = 67 (%)	
Age: (years) ± SD		28.2 ± 8.7	27.2 ± 5.7	.46^*ª*^
Ethnicity	Caucasian	35 (66)	37 (55)	.48^b^
	African American	5 (9)	5 (8)	
	Hispanic	4 (8)	9 (13)	
	Asian	6 (11)	7 (10)	
	Other/Unknown	4 (6)	9 (13)	
Parity	0	36 (67)	38/65 (59)	.06^b^
	1	13 (24)	8/65 (12)	
	2	2 (4)	13/65 (20)	
	>2	3 (6)	6/65 (9)	
Tobacco	None	41 (76)	46 (69)	.10^b^
	<1 ppd	12 (23)	11 (16)	
	1 ppd	1 (2)	8 (12)	
	2 ppd	0 (0)	2 (3)	
Coitarche: (years) ± SD		16.6 ± 2.0	16.5 ± 2.2	.81^a^
No. of Partners	<5	25/49 (51)	22/58 (38)	.17^b^
	>5	24/49 (49)	36/58 (62)	
History of STIs	None	39/51 (77)	44/65 (68)	.92^b^
	CT	3/51 (6)	7/65 (11)	
	GC	0/51 (0)	1/65 (2)	
	Genital Herpes	2/51 (4)	3/65 (5)	
	Genital Warts	2/51 (4)	3/65 (5)	
	PID	1/51 (2)	1/65 (2)	
	More than 1	4/51 (8)	6/65 (9)	
BCM	None	8/52 (15)	1/66 (2)	.01^b^
	Condoms	7/52 (14)	6/66 (9)	
	Oral Contraceptive Pills	31/52 (60)	44/66 (67)	
	Depo-Provera	2/52 (4)	10/66 (15)	
	NuvaRing	1/52 (2)	1/66 (2)	
	Patch	3/52 (6)	0 (0)	
	BTL	0/52(0)	3/66 (5)	
	Copper IUD	0/52 (0)	1/66 (2)	

Denominators may vary due to missing data.

^*ª*^ Student's *t*-test,

^b^ Chi-square.

**Table 2:** Univariate logistic regression of time interval and CIN 2,3 in LEEP.

Time interval (categorical)	CIN 2,3 in LEEP	
	OR (95% CI)	*P*
<3 months	1.0 (Reference)	—
3–5 months	0.63 (0.27–1.44)	.27
>5 months	0.47 (0.18–1.21)	.12

**Table 3:** Univariate logistic regression with birth control method and CIN 2,3 in LEEP.

Variable	CIN 2,3 in LEEP	
	OR (95% CI)	*P*
Birth control method		
None/non-hormonal	1.0 (Reference)	—
Estrogen + Progesterone	1.83 (0.73–4.58)	0.20
Progesterone only	7.59 (1.34–43.2)	0.02

**Table 4:** Multivariate Regression of Time Interval and CIN 2,3 controlling for Birth Control Method.

Variable	CIN 2,3 in LEEP	
	OR (95% CI)	*P*
Time interval (categorical)		
<3 months	(Reference)	—
3–5 months	0.66 (0.27–1.59)	0.35
>5 months	0.39 (0.14–1.07)	0.07
